# Lethal effect of blue light on strawberry leaf beetle, *Galerucella grisescens* (Coleoptera: Chrysomelidae)

**DOI:** 10.1038/s41598-017-03017-z

**Published:** 2017-06-02

**Authors:** Masatoshi Hori, Ayako Suzuki

**Affiliations:** Graduate School of Agricultural Science, Tohoku University, Sendai, 980-0845 Japan

## Abstract

In a previous study, we found that blue-light irradiation kills insects such as fruit flies, mosquitos, and flour beetles. However, the lethal effects of blue light on coleopteran field crop pests have not been investigated. Chrysomelidae, a major family in phytophagous beetles, includes many species of crop pests. We investigated the lethal effect of blue light on chrysomelid beetles by examining the mortality of the strawberry leaf beetle *Galerucella grisescens* irradiated with different wavelengths of blue light during the non-mobile egg or pupal stage by using light-emitting diodes. Fifty to seventy percent of beetles irradiated with 407, 417, 438, or 465-nm lights at 15 × 10^18^ photons·m^−2^·s^−1^ during the egg stage died before hatching; ca. 90% of hatchlings irradiated with 438-nm light during the egg stage died before eclosion; and 35–55% of beetles irradiated with 407, 417, 454, and 465-nm lights at the same intensity during the pupal stage died before eclosion. Field crop pests are considered to have high tolerance to blue light because they are usually exposed to sunlight in their natural habitats. However, this study suggests that blue light can kill some field crop as well as household insect pests.

## Introduction

The toxicity of ultraviolet (UV) light has been reported in various insect pests^1–9^. However, the lethal effects of blue light on insects remained unknown until recently. In a previous paper, we revealed, for the first time, that short-wavelength visible light (blue light, 400–500 nm) can kill some species of insects such as fruit fly, mosquito, and flour beetle^10^. However, knowledge of the lethal effects of blue light on field crop insect pests is still lacking. In the previous paper, we have revealed that tolerance to blue-light irradiation differs among insect species and hypothesized that insect tolerance to blue-light irradiation is closely related to the light exposure experienced in their natural habitat. If this hypothesis is true, blue light may exhibit no lethal effects on field crop insect-pests.

Chrysomelidae is a major family of herbivorous coleopteran insects and includes many species of agricultural pests. Strawberry leaf beetle, *Galerucella grisescens* (Joannis), is a chrysomelid beetle that feeds on leaves of strawberry and polygonaceous plants^11^. The strawberry leaf beetle is an appropriate model species for chrysomelid beetle experiments because it is easily reared and much knowledge about its ecology has been accumulated^12^. In coleopteran insects, the lethal effect of blue light on the pupa of confused flour beetle, *Tribolium confusum* Jacquelin du Val, has been revealed^10^. However, the lethal effect of blue light varies by species even if they belong to the same order. Thus, the tolerance to blue light differs significantly between the fruit fly, *Drosophila melanogaster* Meigen, and the house mosquito, *Culex pipiens molestus* (Forskål), although they belong to the same order Diptera^10^. Irradiation with 417-nm light above 10 × 10^18^ photons·m^−2^·s^−1^ is required for killing efficiently mosquito pupae, whereas 95% of fly pupae are killed by irradiation with 467-nm light at 3 × 10^18^ photons·m^−2^·s^−1^.

Agricultural insect pests are usually controlled by chemical substances such as insecticides. However, physical pest control is attracting attention as a clean pest management method^13^. In recent years, utilization of light for pest control has been particularly studied with the development and popularization of light-emitting diodes (LEDs)^14^. This study aims to explore the possible lethal effects of blue light on insects as a control technique of field crop insect pests. In open or large spaces such as crop fields or greenhouses, adults and larvae of many species of insect pests including chrysomelid beetles can easily move elsewhere. For these reasons, eggs and pupae are preferred targets of continuous irradiation with blue light. Therefore, in this study, we investigated the lethal effect of blue-light irradiation at the egg and pupal stages by using LEDs.

## Results

### Experiment 1: Lethal effects of blue-light irradiation during the egg stage

**Figure 1:**
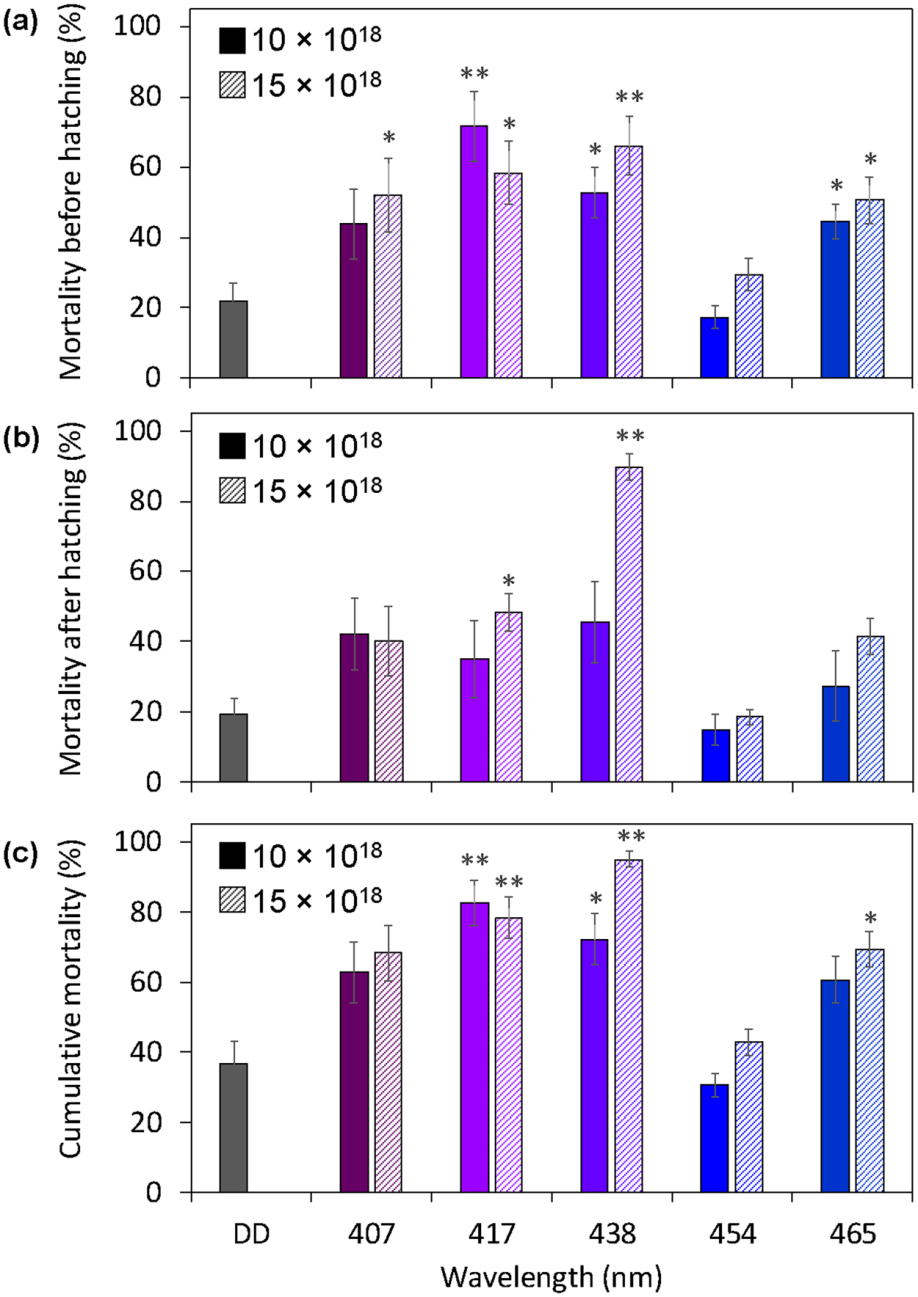
Mortality of beetles that were irradiated with blue light during the egg stage. (**a**) Mortality of beetles that died before hatching. (**b**) Mortality of beetles that died after hatching. (**c**) Cumulative mortality of beetles that died before eclosion. Data are means ± standard error (SE). Inset numbers (10 and 15 × 10^18^) indicate photon flux density (photons·m^−2^·s^−1^). Asterisks above the bars indicate significant differences between the treatments with irradiation and dark conditions (Steel test: *P < 0.05, **P < 0.01). DD indicates dark conditions. Nine replications (20 eggs/replicate) were conducted.

First, we investigated the lethal effect of irradiation with five different wavelengths of blue light during the egg stage. Continuous irradiation with wavelengths of 407, 417, 438, and 465 nm at 10 or 15 × 10^18^ photons·m^−2^·s^−1^ (The actual measured values of the number of photons are shown in Supplementary Table 5) significantly increased egg mortality of the beetles compared with their mortality under dark conditions (DD) (Fig. 1a; Supplementary Table 1). Fifty to seventy percent of the beetles irradiated with these wavelengths of light at 15 × 10^18^ photons·m^−2^·s^−1^ died before hatching. The larvae hatched from eggs irradiated with 438-nm blue light at 15 × 10^18^ photons·m^−2^·s^−1^ showed significantly high mortality compared to the larvae hatched under DD, and approximately 90% of larvae died before eclosion (Fig. 1b; Supplementary Table 2). The increase in the lethal effects of other wavelengths was nominal with increasing number of photons. Cumulative mortalities of the beetles that died before eclosion reached 95% when the beetles were irradiated with 438-nm blue light at 15 × 10^18^ photons·m^−2^·s^−1^ during the egg stage (Fig. 1c; Supplementary Table 3). Irradiation with 407-, 417-, and 465-nm blue lights at 15 × 10^18^ photons·m^−2^·s^−1^ exhibited approximately 70 to 80% cumulative mortality. Cumulative mortality of the beetles irradiated with 454-nm blue light was similar to that under DD although this wavelength is between 438 nm and 465 nm, two wavelengths with a significant lethal effect on the beetles. Among the tested wavelengths, the highest mean temperature of 27.0 °C was measured inside petri dishes irradiated with 465-nm light (Table 1). The lowest mean temperature of 23.5 °C was detected inside petri dishes under DD. There was no association between the temperature inside the petri dish and mortality.

**Table 1:** Temperatures inside petri dishes irradiated with blue light at 15 × 10^18^ photons·m^−2^·s^−1^ in Experiment 1 and 2.

Wavelength (nm)	Temperature (°C)^a^	
	Experiment 1	Experiment 2
407	25.1 ± 0.01	25.6 ± 0.01
417	26.7 ± 0.03	27.6 ± 0.03
438	25.7 ± 0.02	26.6 ± 0.01
454	26.5 ± 0.01	27.6 ± 0.02
465	27.0 ± 0.03	28.6 ± 0.02
DD^b^	23.5 ± 0.01	23.1 ± 0.01

^a^Mean value of the temperatures measured from 12 h to 24 h after the start of irradiation at 5-min intervals using a button-type temperature logger. ^b^DD indicates dark conditions.

### Experiment 2: Lethal effects of blue-light irradiation during the pupal stage

**Figure 2:**
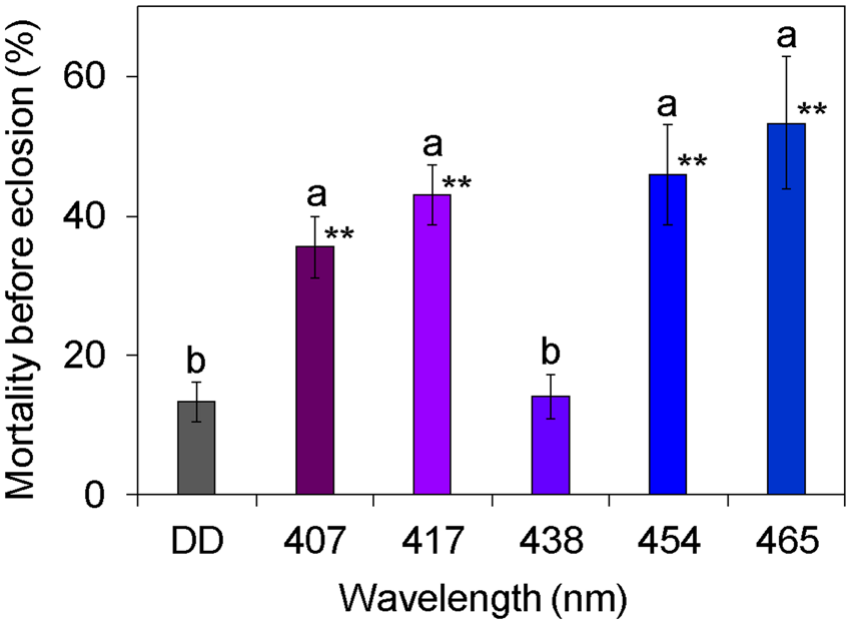
Mortality of beetles that were irradiated with blue light at ca. 15 × 10^18^ photons·m^−2^·s^−1^ during the pupal stage and died before eclosion. Data are means ± standard error (SE). Asterisks at the upper right of the bars indicate significant differences between treatments with irradiation and dark conditions (Steel test: *P < 0.05, **P < 0.01). Different lowercase letters (**a**,**b**) above the bars indicate significant differences (Steel–Dwass test, *P* < 0.05). DD indicates dark conditions. Nine replications (15 pupae/replicate) were conducted.

Continuous irradiation with blue light during the pupal stage also exhibited lethal effects on the beetles (Fig. 2). Irradiation with wavelengths of 407, 417, 454, and 465 nm at 15 × 10^18^ photons·m^−2^·s^−1^ (The actual measured values of the number of photons are shown in Supplementary Table 6) significantly increased pupal mortality of the beetles compared with their mortality under DD. The mortality of beetles irradiated with these wavelengths of light was approximately 40 to 50%, whereas that under DD was 13%. The beetles irradiated with 407- and 417-nm lights died due to weak eclosion failure compared to those irradiated with 454- and 465-nm lights (Supplementary Table 4; Supplementary Figure 1). Irradiation with 438-nm light during the pupal stage was not lethal to beetles although this wavelength showed the highest lethal effect during irradiation at the egg stage (Fig. 2). Among the tested wavelengths of light, the highest mean temperature of 28.6 °C was detected inside petri dishes irradiated with 465-nm light (Table 1); the temperature inside petri dishes under DD was the lowest, with the mean temperature of 23.1 °C. There was no association between the temperature inside the petri dish and mortality.

## Discussion

Our previous study showed that blue light is lethal to household pests such as *Drosophila melanogaster*, *Culex pipiens molestus*, and *Tribolium confusum* ^10^. This study revealed that blue light was also lethal to field crop pests. In Japan, the average temperature in August is 27–29 °C. Therefore, the temperature inside petri dishes irradiated with blue light was not high enough to kill the beetles. In addition, there was no correlation between the temperature inside petri dishes and mortality. That is, the cause of death was not the heat from blue-light irradiation. Although *D. melanogaster* lives in both outdoor and indoor environments, it occupies dark places until adult emergence. *C. pipiens molestus* usually lives in water with dim light until adult emergence and the adult prefers indoor places without sunlight. *T. confusum* lives inside stored grain products until adult emergence, and the adult lives inside or on stored grain products in indoor environments. In contrast, the strawberry leaf beetle spends its entire life cycle in the outdoor environments with sunlight. The photon flux density of the blue light (wavelength: 400–500 nm) in direct sunlight in the field associated with our laboratory (Sendai, Japan; 38°N, 140°E) was ca. 25 × 10^18^ photons·m^−2^·s^−1^ (cloudless sky; September 27, 2015; 11:30 AM). Given that approximately 70 to 90% of strawberry leaf beetles died after continuous irradiation with blue lights at 15 × 10^18^ photons·m^−2^·s^−1^ during egg stage, it has been hypothesised that a large number of beetles die from continuous irradiation of direct sunlight. It is likely that the eggs and pupae are not actually exposed to direct sunlight because they are shaded by plant leaves. The beetles lay their eggs and pupate on the underside of host plant leaves^15^. The transmittance of blue light (400–500 nm) through the leaves of their host plant *Rumex obtusifolius* measured using a high-resolution spectrometer was only 0.013–0.051%. Therefore, it is considered that the eggs and pupae are not exposed to lethal intensities of blue light. Our preliminary experiments using *D. melanogaster* pupae have shown that the middle- and long-wavelength visible light (500–740 nm) did not inhibit the lethal effect of blue light on insects and continuous irradiation was important for the lethal effect of blue light on insects. The actual sunshine duration in Japan is on average approximately 5 h, with the maximum of 14 h^16^. In addition, outdoor insects can easily avoid direct sunlight by hiding behind objects such as plants. Therefore, it is thought that outdoor insects such as the strawberry beetle can live in open fields because they are not exposed to direct sunlight for many hours or even entire day.

Our findings indicate that blue-light irradiation is useful for controlling some species of field crop pests as well as household pests. Since adults and larvae of insects in open or large spaces such as crop fields or greenhouses can easily move and thus avoid irradiation, the continuous irradiation with blue light should target the eggs and pupae. However, the eggs and pupae of insects usually grow on the underside of the leaves or stems even in species that live on plant surfaces during egg and/or pupal stages. Therefore, it is necessary to efficiently irradiate the targets with blue light by using reflective materials such as reflective mulching films. However, since excess light^17–22^ and/or continuous light^23^ cause damages to plants, it is important to examine the influence of blue-light irradiation on target crop when using blue light for crop pest management. Recently, white, blue, and/or red LED devices have been introduced in agriculture, in particular in greenhouses and plant factories. Field crop pests such as leaf beetles easily invade greenhouses and cause damage to crops. Therefore, the introduction of blue LED devices is recommended to control the insect pests in the greenhouses where we can easily control the light environment; for example, the blue LED can be easily incorporated into the supplemental lighting devices used for crop growth. In contrast, excess light during night may influence the behaviour and/or physiology of surrounding plants and animals although the intensities of these lights are lower than the intensity of direct sunlight. Therefore, the possible risks of light pollutions should be considered when applying excess light at night.

It has been revealed that highly toxic wavelengths of visible light are species-specific in insects^10^. This study showed that the effective wavelengths against the beetles were different between the eggs and pupae. That is, 438 nm exhibited the highest toxic effect during the irradiation at the egg stage but the lowest effect during the irradiation at the pupal stage. Contrary to this, 454 nm light was the least toxic during the irradiation at the egg stage and relatively highly toxic during the irradiation at the pupal stage. These results indicate that effective wavelengths vary according to the insect growth stage as well as insect species. UVC (100–280 nm) and UVB (280–315 nm) cause direct damage to DNA by inducing the formation of DNA lesions^24^. In contrast, blue light and UVA (315–400 nm) do not directly damage DNA because the maximum absorption spectrum of DNA ranges from 260 to 265 nm and blue light is not absorbed by native DNA^25–27^. However, it is known that UVA indirectly damages lipids, proteins, and DNA by enhancing the production of reactive oxygen species (ROS)^28–30^, and increases in oxidative stress, molecular-level responses to the stress, and damage caused by UVA irradiation have been shown in some species of insects^31–33^. Blue-light irradiation also injures organisms by stimulating the production of ROS. Mammalian retinas are severely damaged by ROS produced by blue-light irradiation^34, 35^. It is likely that the lethal effect of blue light on insects is caused by the production of ROS, because the effective wavelength is species-specific and growth-stage-specific and not always associated with the amount of delivered photon energy. Therefore, we hypothesized that ROS produced by absorption of specific wavelengths of blue light in species-specific and growth-stage-specific chromophores or photosensitizers in insect tissues damages the tissues and kills the insects.

Currently, we are studying the mechanisms of lethal effects of blue light on insects. In the preliminary experiments, we confirmed that the amount of H_2_O_2_ in the whole body of *Drosophila* pupae was increased by blue-light irradiation and showed similar specificity of wavelengths between H_2_O_2_ production and lethal effects. In addition, we confirmed that the growth of cultivated cells of *Drosophila* embryos was suppressed by blue-light irradiation.

In the present study, we investigated the lethal effect of only blue lights because the photon flux densities of green (530 nm), yellow (590 nm), and red (660 nm) lights were less than 10 × 10^18^ photons·m^−2^·s^−1^ at the maximum power. It is thought that the lethal effects of wavelengths of light longer than 530 nm are negligible because these wavelengths were not lethal to *Drosophila* pupae^10^ and did not suppress the growth of *Drosophila* cells in the preliminary experiments.

The wavelengths that are effective against the target growth stage of a target insect should be investigated when using blue light for pest control because the effective wavelength of blue light is species-specific and growth-stage-specific. The wavelength of around 440 nm, which is considered appropriate for irradiation during the egg stage, and that of around 470 nm, which is commonly used in blue LED lights and exhibited a relatively high effect on both the eggs and pupae, are promising wavelengths for controlling the strawberry leaf beetle. In the near future, we will examine the efficacies of LED lights with these wavelengths against beetles in field tests.

## Methods

### Insects

Eggs and pupae of the strawberry leaf beetle *Galerucella grisescens* (Joannis) collected at the experimental field of Tohoku University and reared for successive generations in a constant-temperature room at 24 ± 1 °C under a photoregime of 16L:8D were used in the experiments. The beetles were reared on leaves of *Rumex obtusifolius* L. collected from the experimental field.

### LED light radiation

LED lighting units (IS-mini^®^, ISL-150 × 150 Series; CCS Inc., Kyoto, Japan; light emission surface: 150 × 150 mm; 360 LEDs were equally arranged on a panel; LED type: φ 3-mm plastic mould) with power supply units (ISC-201-2; CCS Inc.) were used for blue light radiation. Insects were irradiated with LED light in a multi-room incubator (LH-30CCFL-8CT; Nippon Medical & Chemical Instruments Co., Ltd., Osaka, Japan). The emission spectrum was measured using a high-resolution spectrometer (HSU-100S; Asahi Spectra Co., Ltd., Tokyo, Japan; numerical aperture of the fibre: 0.2). Emission spectra used in the experiments are compared in Supplementary Figure 2. The number of photons (photons·m^−2^·s^−1^) was measured using the spectrometer in a dark room and was adjusted using the power-supply unit. The distance between the light source and the spectrometer sensor during the measurements was 220 mm which was the same as that between the insects and the light source in the incubator. Because the insects were irradiated through a polystyrene lid, the same lid was placed between the light source and the sensor during the measurements. The distance between the lid and the light source during the measurements was 205 mm, which was the same as those in the incubator. Five LED panels with different wavelengths, 407, 417 438, 454, and 465 nm, were used for the tests. Polystyrene petri dishes containing insects were placed directly under the light source during irradiation. We verified that the upper surfaces of the petri dishes were irradiated homogeneously by measuring the numbers of photons. In addition, we assumed that temperature changes caused by the light source would not affect survival of the insects because LED light emits little heat. To test this assumption, we measured the temperature inside the petri dishes from 12 h to 24 h after the start of irradiation at 5-min intervals using a button-type temperature logger (3656, Hioki E. E. Co., Ueda, Japan). The temperatures during the irradiation with all the wavelengths of light used in the experiments and under DD were measured under the same conditions as described in Experiment 1 and 2.

### Experiment 1: Lethal effects of blue-light irradiation during the egg stage

Twenty eggs were collected within 24 h after deposition from seven- to eight-day-old adult female beetles and placed on a sheet of filter paper (ADVANTEC MFS, Inc., Dublin, CA, USA; No. 1, 70 mm in diameter) impregnated with 750 μL of water in a polystyrene petri dish (55 mm in diameter, 15 mm in height). The petri dish was sealed with a layer of Parafilm around the edge, placed in the multi-room incubator, and irradiated continuously with LED light at 0 (DD), 10 × 10^18^, and 15 × 10^18^ photons·m^−2^·s^−1^ during the egg stage at 25 ± 1 °C. The actual measured values of the number of photons are shown in Supplementary Table 5. The number of hatchlings in the petri dish was counted every 24 h. Hatchlings were picked up by a fine calligraphy brush and placed on a sheet of filter paper (ADVANTEC, No. 1, 50 mm in diameter) impregnated with 450 μL of water in a polystyrene petri dish (50 mm in diameter, 9 mm in height). The eggs that did not hatch within 10 days were removed from the multi-room incubator, placed under continuous DD at 25 ± 1 °C for 10 days, and confirmed dead. The petri dish containing the hatchlings was sealed with a layer of Parafilm around the edge. The hatchlings in the petri dishes were reared on leaves of *R. obtusifolius* at 25 ± 1 °C under continuous DD. The leaves of *R. obtusifolius* were replaced with new ones every 2 days. Nine replications (i.e., petri dishes) were conducted for each wavelength and dose. The mortality of beetles that died before hatching and the mortality of beetles that hatched and died before adult eclosion were investigated. Almost all of the beetles that pupated could become adults.

### Experiment 2: Lethal effects of blue-light irradiation during the pupal stage

Fifteen prepupae were collected from the stock cultures and placed in a polystyrene petri dish (55 mm in diameter, 15 mm in height). The petri dish was sealed with a layer of Parafilm around the edge, placed in the multi-room incubator, and irradiated continuously with LED light at 0 (DD) and 15 × 10^18^ photons·m^−2^·s^−1^ for 10 days at 25 ± 1 °C. The actual measured values of the number of photons are shown in Supplementary Table 6. The number of emerging adults was counted 10 days after the start of the irradiation. Nine replications (i.e., petri dishes) were performed for each wavelength and dose. The pupae that did not emerge within 10 days were removed from the multi-room incubator, placed under continuous dark conditions at 25 ± 1 °C for 10 days, and confirmed dead. The states of the beetles that died before emergence were also investigated (i.e., weak eclosion failure, strong eclosion failure, pupal death, prepupal death; Supplementary Figure 1).

### Statistical analyses

All experimental data were statistically analysed with a Kruskal-Wallis test followed by the Steel-Dwass test and Steel test.

## Electronic supplementary material

Supplementary Information 
