# Supplementary material for: Lethal effect of blue light on strawberry leaf beetle, Galerucella grisescens (Coleoptera: Chrysomelidae)
Source: Sci Rep. 2017 Jun 2;7:2694. doi: 10.1038/s41598-017-03017-z (PMC5457428; doi:10.1038/s41598-017-03017-z)
Supplement: Supplementary file 1 — Supplementary Information [file 41598_2017_3017_MOESM1_ESM.pdf]

Lethal effect of blue light on strawberry leaf beetle, *Galerucella grisescens* (Coleoptera: Chrysomelidae)

Masatoshi Hori* & Ayako Suzuki

Graduate School of Agricultural Science, Tohoku University, Sendai 980-0845, Japan

*Correspondence: hori@tohoku.ac.jp

Supplementary Table 1. Mortality of beetles that were irradiated with blue light during the egg stage and died before hatching.

Wavelength (nm)	Mean mortality of beetles before hatching ± SE (%)							
	Photon flux density (× 10^{18} photons▪m^{-2}▪s^{-1})							
	10				15			
407	43.89	±	9.99	ab	52.22	±	10.54	abc
417	71.67	±	10.03	a	58.33	±	8.90	ab
438	52.78	±	7.17	a	66.11	±	8.32	a
454	17.22	±	3.34	b	29.44	±	4.60	bc
465	44.44	±	4.89	a	50.56	±	6.74	ab
DD	20.56	±	4.44	b	20.56	±	4.44	c

Values followed by different letters (a, b, c) in the same row are significantly different (Steel-Dwass test, $P < 0.05$). DD indicates dark conditions.

Supplementary Table 2. Mortality of beetles that were irradiated with blue light during the egg stage and died after hatching.

Wavelength (nm)	Mean mortality of beetles after hatching ± SE (%)	
	Photon flux density (× 10^{18} photons▪m^{-2}▪s^{-1})	
	10	15
407	42.13 ± 10.28	40.11 ± 9.82 bc
417	35.00 ± 10.97	48.28 ± 5.47 b
438	37.00 ± 11.05	89.67 ± 3.66 a
454	15.55 ± 4.09	18.54 ± 2.20 c
465	27.27 ± 10.05	41.29 ± 5.06 b
DD	21.54 ± 5.11	21.54 ± 5.11 bc

Values followed by different letters (a, b, c) in the same row are significantly different (Steel-Dwass test, $P < 0.05$). No significant difference was obtained at 10 × 10^{18} photons▪m^{-2}▪s^{-1} according to the Kruskal-Wallis test ($P > 0.05$). DD indicates dark conditions.

Supplementary Table 3. Cumulative mortality of beetles that were irradiated with blue light during the egg stages and died before eclosion.

Wavelength (nm)	Mean cumulative mortality ± SE (%)							
	Photon flux density (× 10^{18} photons·m^{-2}·s^{-1})							
	10				15			
407	62.78	±	8.74	abc	68.33	±	7.99	abc
417	82.78	±	6.46	a	78.33	±	5.89	b
438	72.22	±	7.22	ab	95.00	±	2.20	a
454	30.56	±	3.38	c	42.78	±	3.74	c
465	60.56	±	6.64	ab	69.44	±	5.17	b
DD	36.67	±	6.40	bc	36.67	±	6.40	c

Values followed by different letters (a, b, c) in the same row are significantly different (Steel-Dwass test, $P < 0.05$). DD indicates dark conditions.

Supplementary Table 4. States of dead beetles irradiated with blue light during the pupal stage (For explanation of the states see Supplementary Figure 1).

Wavelength (nm)	Mean percentages of each state of dead beetle ± SE						
	Weak eclosion failure		Strong eclosion failure	Pupal death		Prepupal death	
407	45.99 ± 10.85	ab	36.07 ± 10.09	11.73 ± 5.67	ab	6.21 ± 3.24	ab
417	52.22 ± 8.12	a	27.96 ± 8.66	15.45 ± 5.55	ab	4.37 ± 3.02	ab
438	10.37 ± 7.21	b	18.52 ± 11.26	5.93 ± 4.07	b	65.19 ± 12.99	a
454	17.37 ± 7.77	b	32.11 ± 9.81	31.02 ± 10.42	ab	19.50 ± 6.28	ab
465	13.22 ± 4.56	ab	46.77 ± 7.86	39.22 ± 5.25	a	0.79 ± 0.79	b
DD	8.33 ± 5.69	ab	24.00 ± 10.97	45.00 ± 11.93	ab	22.67 ± 11.98	ab

Values followed by different letters (a, b) in the same row are significantly different (Steel-Dwass test, $P < 0.05$). No significant difference was obtained for the strong eclosion failure according to the Kruskal-Wallis test ($P > 0.05$). DD indicates dark conditions.

Supplementary Table 5. Actual measured values of the photon flux density in the experiment measuring lethal effects of blue-light irradiation during the egg stage.

Wavelength (nm)	Mean actual measured values ± SE (× 10^{18} photons·m^{-2}·s^{-1})*	
	Set values (× 10^{18} photons·m^{-2}·s^{-1})	
	10	15
407	10.12 ± 0.02	16.10 ± 0.03
417	10.08 ± 0.01	15.41 ± 0.02
438	12.02 ± 0.04	15.31 ± 0.03
454	10.42 ± 0.03	15.11 ± 0.02
465	10.01 ± 0.03	14.53 ± 0.01

*Mean value of the measurements conducted five times before and after each irradiation test. Photon flux density was measured using a high-resolution spectrometer (HSU-100S; numerical aperture of the fibre: 0.2).

Supplementary Table 6. Actual measured values of the photon flux density in the experiment measuring lethal effect of blue-light irradiation during the pupal stage.

Wavelength (nm)	Mean actual measured values ± SE (× 10^{18} photons▪m^{-2}▪s^{-1})*
407	15.39 ± 0.17
417	15.22 ± 0.01
438	15.38 ± 0.02
454	15.03 ± 0.01
465	14.88 ± 0.02

*Mean value of the measurements conducted five times before and after each irradiation test. Photon flux density was measured using a high-resolution spectrometer (HSU-100S; numerical aperture of the fibre: 0.2).

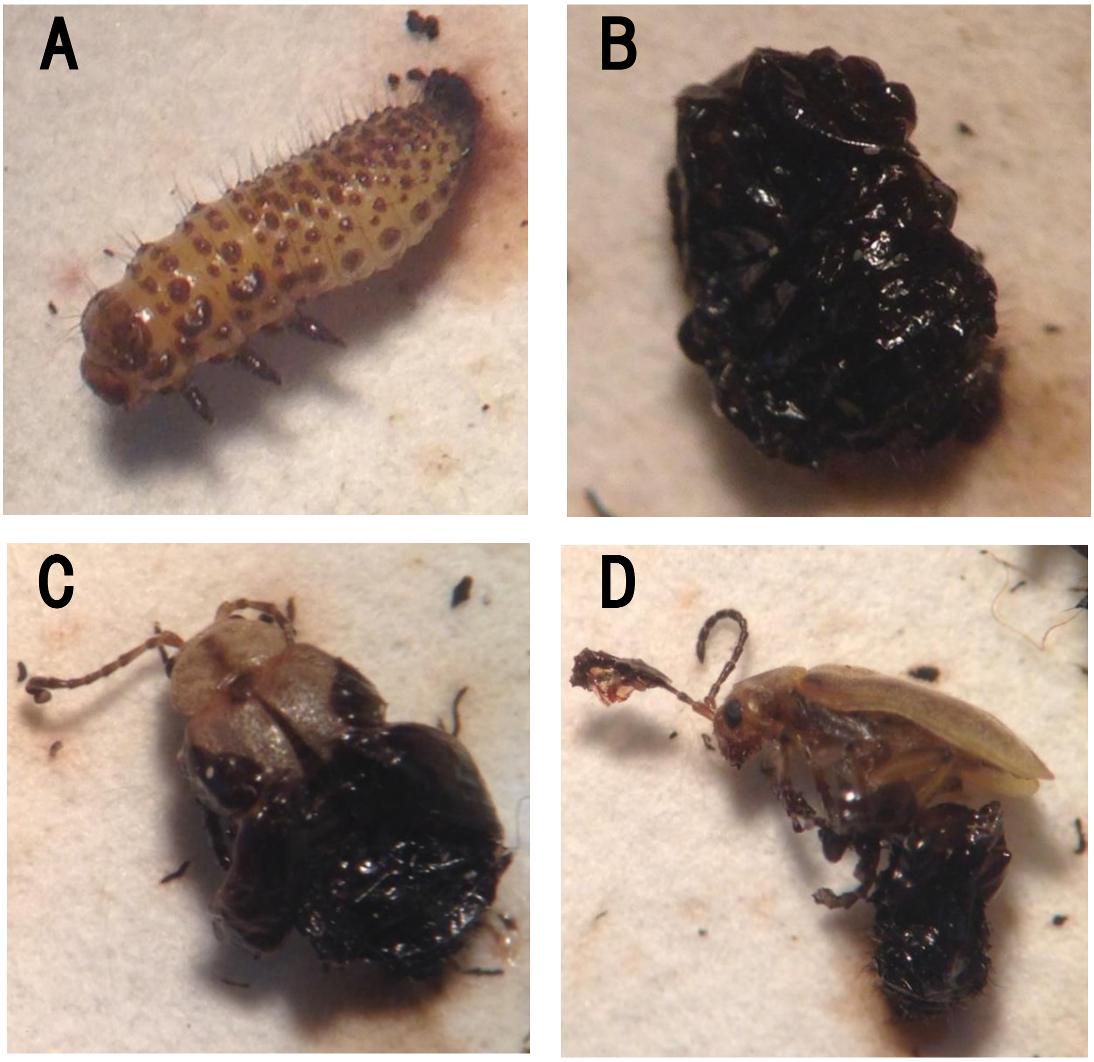

Supplementary Figure 1. The states of dead beetles irradiated with blue light during the pupal stage (See Supplementary Table 4). (A) Prepupal death. The beetle died at the prepupal stage. (B) Pupal death. The beetle died at the pupal stage. (C) Strong eclosion failure. The beetle died in the early-to-middle eclosion period. (D) Weak eclosion failure. The beetle died in the late eclosion period (The beetle could not emerge because its legs were caught in its shell).

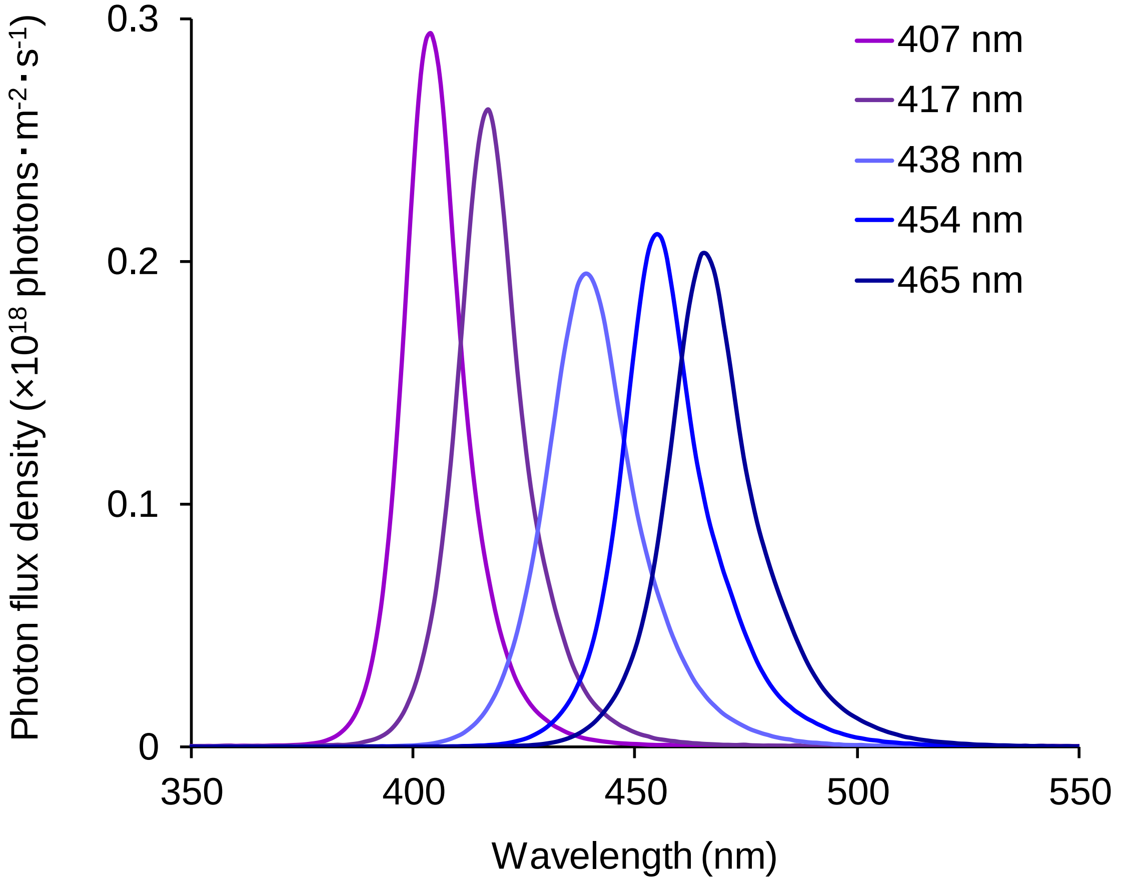

Supplementary Figure 2. Emission spectra of LED lighting units used in the experiments.
